# Exposure Routes and Health Risks Associated with Pesticide Application

**DOI:** 10.3390/toxics10060335

**Published:** 2022-06-19

**Authors:** Muyesaier Tudi, Hairong Li, Hongying Li, Li Wang, Jia Lyu, Linsheng Yang, Shuangmei Tong, Qiming Jimmy Yu, Huada Daniel Ruan, Albert Atabila, Dung Tri Phung, Ross Sadler, Des Connell

**Affiliations:** 1 Key Laboratory of Land Surface Pattern and Simulation, Institute of Geographical Sciences and Natural Resources Research, Chinese Academy of Sciences, No. 11 Datun Road, Beijing 100101, China; m.tudi@griffith.edu.au (M.T.); liwang@igsnrr.ac.cn (L.W.); lvjia@nieh.chinacdc.cn (J.L.); yangls@igsnrr.ac.cn (L.Y.); tongsm.19b@igsnrr.ac.cn (S.T.); 2 School of Medicine, Griffith University, 170 Kessels Road, Nathan, Brisbane, QLD 4111, Australia; d.phung@griffith.edu.au (D.T.P.); ross.sadler@griffith.edu.au (R.S.); 3 Foreign Environmental Cooperation Center, Ministry of Ecology and Environment, Beijing 100035, China; li.hongying@fecomee.org.cn; 4 China CDC Key Laboratory of Environment and Population Health, National Institute of Environmental Health, Chinese Center for Disease Control and Prevention, No. 29 Nanwei Road, Beijing 100050, China; 5 School of Engineering and Built Environment, Nathan Campus, Griffith University, Brisbane, QLD 4111, Australia; jimmy.yu@griffith.edu.au; 6 Environmental Science Program, Division of Science and Technology, Beijing Normal University-Hong Kong Baptist University United International College, 2000 Jintong Road, Tangjiawan, Zhuhai 519087, China; hruan@uic.edu.cn; 7 Department of Biological, Environmental & Occupational Health Sciences, School of Public Health, University of Ghana, Legon, Accra P.O. Box LG13, Ghana; albert.atabila@gmail.com; 8 School of Environment and Science, Griffith University, 170 Kessels Road, Nathan, Brisbane, QLD 4111, Australia; d.connell@griffith.edu.au

**Keywords:** human health risk assessment, occupational health and safety, pesticide application methods, pesticide exposure

## Abstract

Pesticides play an important role in agricultural development. However, pesticide application can result in both acute and chronic human toxicities, and the adverse effects of pesticides on the environment and human health remain a serious problem. There is therefore a need to discuss the application methods for pesticides, the routes of pesticide exposure, and the health risks posed by pesticide application. The health problems related to pesticide application and exposure in developing countries are of particular concern. The purpose of this paper is to provide scientific information for policymakers in order to allow the development of proper pesticide application technics and methods to minimize pesticide exposure and the adverse health effects on both applicators and communities. Studies indicate that there are four main pesticide application methods, including hydraulic spraying, backpack spraying, basal trunk spraying, and aerial spraying. Pesticide application methods are mainly selected by considering the habits of target pests, the characteristics of target sites, and the properties of pesticides. Humans are directly exposed to pesticides in occupational, agricultural, and household activities and are indirectly exposed to pesticides via environmental media, including air, water, soil, and food. Human exposure to pesticides occurs mainly through dermal, oral, and respiratory routes. People who are directly and/or indirectly exposed to pesticides may contract acute toxicity effects and chronic diseases. Although no segment of the general population is completely protected against exposure to pesticides and their potentially serious health effects, a disproportionate burden is shouldered by people in developing countries. Both deterministic and probabilistic human health risk assessments have their advantages and disadvantages and both types of methods should be comprehensively implemented in research on exposure and human health risk assessment. Equipment for appropriate pesticide application is important for application efficiency to minimize the loss of spray solution as well as reduce pesticide residuals in the environment and adverse human health effects due to over-spraying and residues. Policymakers should implement various useful measures, such as integrated pest management (IPM) laws that prohibit the use of pesticides with high risks and the development of a national implementation plan (NIP) to reduce the adverse effects of pesticides on the environment and on human health.

## 1. Introduction

Pesticides play an important role in agricultural development as they can reduce the loss of agricultural products and improve the affordable yield and quality of food [1,2,3]. Owing to the urgency to improve food production and control insect-borne diseases, the development of pesticides increased during World War II (1939–1945), and from the 1940s onwards, the increased use of synthetic crop protection chemicals permitted a further increase in food production [4]. Moreover, pesticide production worldwide has increased at a rate of about 11% per annum, from 0.2 million tons in the 1950s to more than 5 million tons by 2000 [5]. Three billion kilograms of pesticides have been consumed worldwide every year [4], while only 1% of total pesticides were effectively used to control insect pests on target plants. The remaining large amounts of pesticides go into or reach non-target plants and environmental media. As a result, pesticide contamination has significantly polluted the environment and caused adverse impacts on human health [6,7,8].

Equipment for appropriate pesticide application is important for application efficiency [9] to minimize the loss of spray solution as well as reduce pesticide residuals in the environment and adverse human health effects due to over-spraying and residues. Humans are directly exposed to pesticides in the workplace [10] and indirectly through environmental media, such as air, water, soil, and the food chain, which may be contaminated with pesticides [11,12]. Dermal, oral, and respiratory routes are the main common pathways by which pesticides enter the human body [13,14]. According to a WHO and UNEP report, worldwide, three million people are poisoned and 200,000 die due to pesticide exposure [15].

Although some measures have been proposed to reduce the adverse effects of pesticides on the environment and human health [16], both acute and chronic toxicity to humans resulting from these substances remain a serious problem. It is predicted that the risk of pesticide exposure will increase worldwide over the next decade, especially in developing countries [17,18]. There has been much work on the need to reduce the use of pesticides but there is little focus on how correct and proper application methods can help to reduce pesticide exposure. Therefore, there is a need to discuss the routes of pesticides exposure and related health problems. The purpose of this paper is to provide a review of state-of-the-art pesticide application methods, the main routes of pesticide exposure and the related human health effects, the basic framework of health risk characterization, and the advantages and disadvantages of different risk characterization methods for the development of strategies by policymakers in relation to the establishment of proper pesticide application technics and methods to minimize pesticide exposure and reduce the health effects on both applicators and communities. The review also provides an up-to-date bibliography for this broad subject.

## 2. Pesticide Application Methods

Pesticide application methods are selected by considering the habits of the target pests, the characteristics of the target site, and the properties of the pesticides [19,20]. The most common pesticide application technics are band spraying, broadcast spraying, drench, foliar, soil injection, space treatment, and spot treatment [19].

Two commonly used technics to uniformly treat crop areas are broadcast and band spraying. Band spraying is a method in which non-selective herbicides are used along fence rows and borders to kill all vegetation. In broadcast spraying, pesticides are uniformly applied to a large area of turf grass either on foot or using motorized equipment [21]. Spray pressure, walking speed, nozzle tip, and height are important factors related to the efficiency of these application methods. The Kentucky Pesticide Education Program (2016) also shows that hand pumping determines the spray pressure. Selecting a suitable constant walking speed and a nozzle tip for the volume of mixed spray enables the proper use of pesticides.

Spot and space treatments are commonly used to treat scattered clumps of weeds or brush. These application methods are efficient ways to treat specific problem areas without treating an entire turf area. Manual sprayers are designed for spot treatments and space treatment areas unsuitable for larger areas [22]. Compared to other sprayers, they are relatively inexpensive and are easy to operate, clean, and store. Adjustable spray guns are also used for lawn care sprays [23].

There are several means by which non-spray pesticide can be applied, including drench, foliar, and soil injection methods. Drenching is a method where pesticides are specifically applied to the root system and move through a plant, foliar is where pesticides are applied directly to the leafy portions of a plant, and soil injection is where pesticides are directly placed on the soil instead of onto a growing plant. The wiper application is used to wipe a non-selective herbicide onto the plant and selectively kill individual weeds. These approaches allow for more efficient use of pesticides and more effective placement, especially against some borers, and eliminate drift.

### 2.1. Hydraulic Sprayers

Hydraulic sprayers range from powered units with multi-nozzle boomd to hand-pumped backpack sprayers [24]. In all cases, pressure from either a pump or compressed gas or air is used to atomize the spray mix at the nozzle [25]. High-pressure pumps are needed to provide good spray coverage for large trees.

### 2.2. Backpack Sprayers

The backpack sprayer is simple and consists of a tank, a pump, a spray wand, and a nozzle [25,26]. It is useful for treating small areas, spot spraying, and covering hard-to-reach locations [27]. The main spray options are broadcast, band, space, and spot treatment [28]. 

### 2.3. Basal Trunk Sprayers

Basal trunk spraying is a pesticide application approach that is easy and quick to apply and requires no special equipment other than a garden sprayer [29]. This method involves thoroughly wetting the lower 5 feet of a tree trunk with a water-soluble pesticide [30]. Pesticides are absorbed through the bark and distributed by the vascular system of the tree. This application method does not harm the tree, as the chemicals do not pollute the soil when applied properly by farmers.

### 2.4. Aerial Sprayers

Helicopters or fixed-wing aircraft are used for spraying pesticides on crops and plantations in large and inaccessible areas [31], such as highlands and special areas where pests and crop diseases occur repeatedly and frequently [32] and when areas are not suitable for ground-based spraying [23]. 

**Figure 1:**
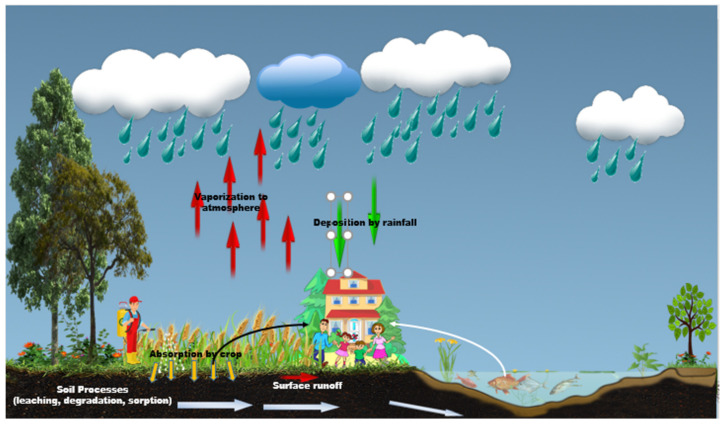
Pesticide behavior in the natural environment in a crop field (by authors).

When pesticides are applied to a target plant by the different application methods, they have the potential to enter the environment [33]. On entering the environment, pesticides can undergo processes such as transfer (or movement) and degradation (Figure 1) [34,35,36]. Pesticides migrate from the target site to other environmental media or non-target plants by transfer processes, including adsorption, leaching, volatilization, spray drift, and runoff. Improper pesticide usage and management and pesticide behavior in the environment lead to environmental pollution, including soil pollution, water pollution, air pollution, and food contamination [33]. Thus, residents and farmers are exposed to pesticides through exposure media, including soil, water, air, and contaminated food.

## 3. Routes of Pesticide Exposure

**Figure 2:**
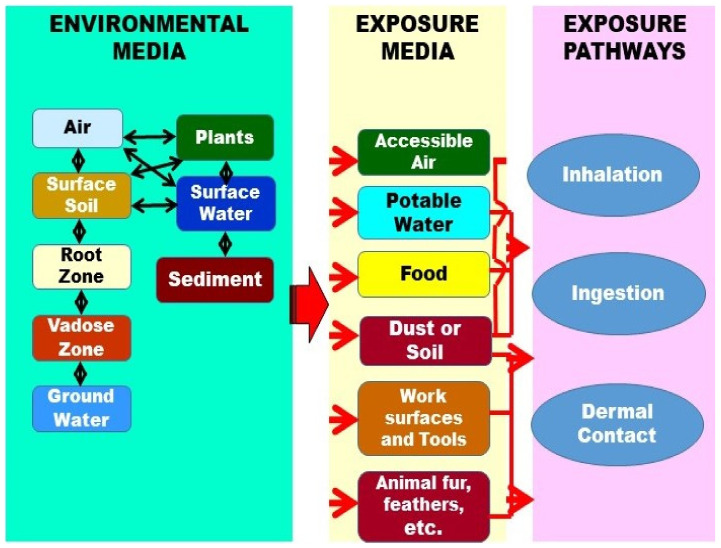
Routes of exposure to pesticides (by authors).

People are not only directly exposed to pesticides in occupational, agricultural, and household activities [37,38,39] through different application methods [40], they are also indirectly exposed to pesticides via contaminated environmental media, including air, water, soil, and food [10,11,41,42,43]. These different types of exposure determine the degree of toxicity of pesticides [6,13,40]. The main ways pesticides come into contact with the human body are through the dermal, oral, and respiratory routes (Figure 2) [10,33,44,45,46,47].

Dermal exposure is the most common and effective exposure route [48,49,50] for farmers who are exposed to pesticides because of splashing, spillage, or spray drift of pesticides [51,52,53], especially when they use pesticides in agriculture or in household activities [1,54,55,56,57]. For example, Wang et al. [56] performed a risk assessment of workers directly dermally exposed to Trinexapac-ethyl (TE) using absorbent paper patches. The exposure intensity (EI) and potential dermal exposure (PDE) for the body sections of workers were obtained and the results showed that the EI of each body section among mixing/loading, hand-held power sprayer, and manual sprayer workers ranged from 7.22 to 73.0 pg·cm^−2^. The maximum EI of TE was found on the hands of manual sprayers, while the minimum EI of TE was recorded on the upper arms of mixing and loading workers. The maximum exposure values for the various body sections were 29% for the chests and backs of mixing/loading workers, 40% for the chests and backs of hand-held power sprayers, and 32% for the thighs of manual sprayers. Taufeeq et al. [3] investigated the contamination of OCPs in River Barandu and carried out a human health risk assessment. The results showed that the ΣOCP levels in sediments ranged between 32.9 and 98.8 ng/g and in water ranged between 0.340 and 0.935 μg/L. Hexachlorocyclohexanes (HCHs) and heptachlor were the most prevalent pesticides in both matrices of the river. The lifetime carcinogenic and non-carcinogenic health risks associated with dermal exposure to the OCP-contaminated river water were considered nominal for the surrounding populations. Han et al. [58] detected the dermal exposure and assessed the risk of pesticide exposure for those involved in the seed-coating process. The results of the study indicated that the levels of chemical exposure varied by the type of work undertaken and the region of a worker’s body exposed. Handling tebuconazole during seed-coating had a low risk, whereas handling carbofuran posed a potential risk to human health.

The most common poisoning route is oral exposure, which causes severe health problems [59,60,61,62,63]. Oral exposure to pesticides can arise when people who produce or use pesticides do not wash their hands before eating or smoking [14,64,65] and the general populace can be subjected to oral exposure when they consume food that is contaminated with pesticides [66,67,68].

Respiratory exposure (via inhalation or breathing) occurs because of the volatile components of pesticides [69,70,71,72] and it is dangerous to workers’ health, especially their nose, throat, and lung tissues if they inhale large amounts of pesticides in the environment through air, water, and soil [33,70,71,72,73]. For example, Yoshida et al. [72] evaluated household exposure to pyrethroids through all exposure pathways and discussed the contribution of the inhalation pathway in Japanese children. The urine excreted first after waking up was collected from subjects aged 6 to 15 years (*n* = 132), and airborne pyrethroids were sampled in the subjects’ bedrooms for 24 h. The contribution rates of the amounts absorbed by inhalation relative to the amounts absorbed via all the exposure pathways tended to decrease in the following order: profluthrin (median 15%) ≈ transfluthrin (14%) > metofluthrin (1%) > bifenthrin (0.1%). Transfluthrin was the most notable pyrethroid as an indoor air pollutant, and residents exposed to Transfluthrin experienced adverse health effects.

## 4. Human Health Effects Related to Pesticide Exposure

**Figure 3:**
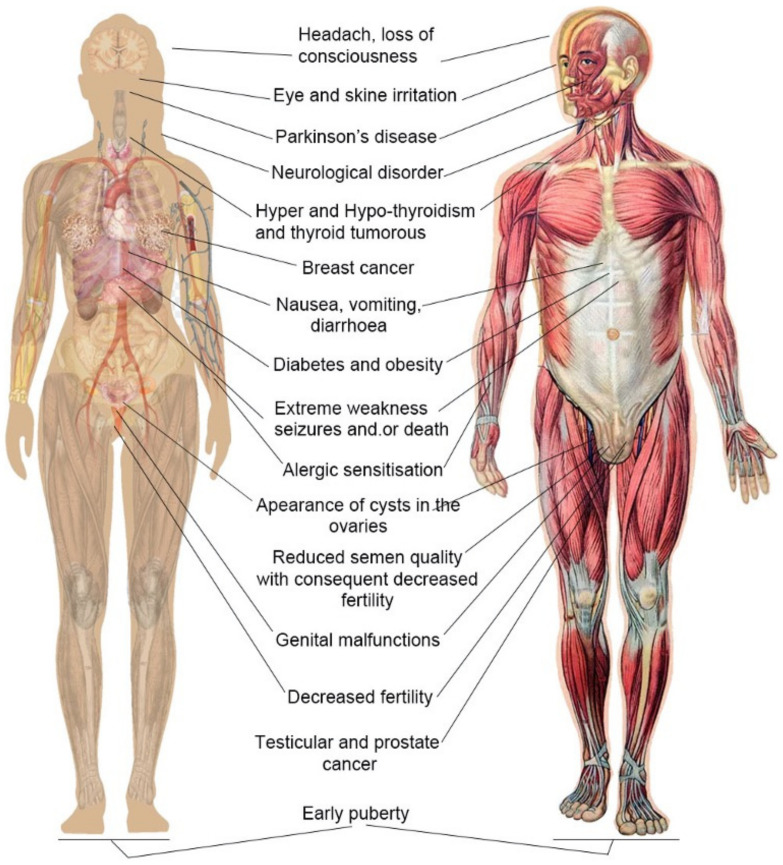
Health problems due to pesticide exposure (by authors).

Owing to pesticides’ important role in agricultural development, there is a heavy dependence on pesticide applications to meet the huge demand for food production by an increasing population. This causes environmental stress and has detrimental health effects on humans worldwide [9,74]. The high-risk groups directly exposed to pesticides are workers, formulators, sprayers, mixers, loaders, and agricultural farm workers [4,75,76]. During manufacturing and formulation, contact with hazardous materials and situations are greatly increased, as the processes involved are not risk-free. Moreover, workers are directly exposed to pesticides via their hands and by inhaling chemicals in the air phase. In industrial settings, workers are also at an increased risk because they need to handle various toxic chemicals, including pesticides, raw materials, toxic solvents, and inert carriers [77]. Humans are also indirectly exposed to pesticides from contaminated soil, air, water, and the food chain [10,11,14]. Humans can be exposed to pesticides in a multitude of ways, both directly and indirectly (Phung et al.) [16], which cause different health effects [78,79] (Figure 3).

People who are both directly and indirectly exposed to pesticides may suffer acute toxic effects, including suicide attempts, mass poisoning from contaminated food, chemical accidents in the industry (WHO, 1990), occupational exposure in the agricultural industry [80,81,82,83], and a number of serious chronic diseases [5,44], including cancer, asthma, diabetes, Parkinson’s disease, leukemia, and cognitive impairment [11,14,83,84]. Many cases of intoxication of farmers, rural workers, and their families during pesticide application in agricultural activities have been identified [85,86,87,88,89,90]. There have also been reports of poisoning and the effects of chemicals on human health from the environment and contaminated food [60,91,92,93,94,95]. According to the WHO, unintentional poisonings kill an estimated 355,000 people globally each year, and poisonings are strongly related to excessive exposure and inappropriate use of toxic pesticides [80].

### 4.1. Acute Toxic Effects

Acute toxic effects occur within a few minutes to several hours after poisoning by pesticides [77,96,97,98]. Poisoning impacts peripheral muscarinic and nicotinic receptors, as well as the central nervous system [99,100,101,102,103]. Some manifestations of a cholinergic crisis include nausea, vomiting, diarrhea, abdominal cramp, urinary incontinence, miosis, salivation, lacrimation, bronchorrhea, bradycardia, hypotension, fasciculation, muscle paralysis, dizziness, confusion, seizures, coma, and respiratory failure [8,100,102,104,105,106]. These effects may occur immediately with exposure to pesticides [107,108,109,110]. Moreover, if life-threatening complications are not properly and immediately treated, death can also occur [111,112,113].

In what follows, some case studies of the acute toxic effects of pesticides on animals and humans are summarized. Mishra et al. [97] assessed the toxic effects of CPF on histopathological changes in pseudobranchial neurosecretory cells (PNSCs) of a neuroendocrine system of the gill region, the optic tectum (OT) and cerebellum, biochemical changes (acetylcholinesterase (AChE) activity and antioxidant markers) in the brain, and associated locomotory behavioral alterations in air-breathing catfish *Heteropneustes fossilis*. The results of the study indicated that acute exposure to CPF for a short duration may induce dysregulation of the neurosecretory activity of PNSCs, altered biochemical activity of brain, and reduced locomotory/swimming performance in fishes. Uçkun et al. [100] investigated the acute toxic effects of Thmx on A. leptodactylus using various biomarkers (acetylcholinesterase, carboxylesterase, glutathione S-transferase, glutathione, superoxide dismutase, glutathione peroxidase, glutathione reductase, and adenosinetriphosphatases). The 96 h LC_50_ value of Thmx was 8.95 mg/L as active ingredient. Thmx has highly toxic effects on crayfish; therefore, they are under threat in the areas where this pesticide is used. Kwon et al. [101] applied a passive dosing format using a silicone O-ring as a reservoir and evaluated its applicability for the determination of the effects of PCP on Daphnia magna. The results of the study showed that the partition coefficient of PCP between methanol and a test medium (log KMeOH:ISO) was 2.1, which enabled the maintenance of a reliable exposure concentration throughout the experiment. In the acute toxicity tests, passive dosing and solvent spiking showed similar EC_50_ values of 576 and 485 μg/L for 24 h, and 362 and 374 μg/L for 48 h, respectively, which overlap with the EC_50_ values of previous studies. Brown et al. [107] determined the acute lethal effects of a 1 h pulse exposure of selected insecticides on adult and juvenile (<72 h old) crimson-spotted rainbowfish *Melanotaenia duboulayi* (Castlenau). The results of the study indicated that temephos and pirimiphos-methyl were toxic to juveniles, with 24 h pulse exposure LC_50_ values of 27 and 15 ug/L, respectively. Of the two OPs, pirimiphos-methyl was the most toxic, with a lethal dose ratio (pulse exposure LC_50_ temephos/pulse exposure LC_50_ pirimiphos-methyl) of 1.8 (95% CL 0.5Ð 6.4). These pulse exposure LC_50_ values represented 40 and 4.5% of the estimated environmental concentrations (EECs) for a 15 cm deep water body, respectively. Chen et al. [108] conducted a series of calorimetric experiments to investigate the toxic effects of beta-cypermethrin (BCP), bensulfuron-methyl (BSM), and prometryne (PM) on *Pseudomonas putida* (*P. putida*). The results of the study indicated that BSM was the most toxic, with an IC_50_ of 19.24 μg/mL against *P. putida*. PM exhibited moderate virulence with an IC_50_ of 27.86 μg/mL, and BCP had the lowest toxicity with an IC_50_ of 39.64 μg/mL. Abss et al. [103] discussed the ability of 18 pesticides to inhibit selective model activities for all major xenobiotic-metabolizing enzymes, namely, CYP1A11/2, 2A6,, 2B6, 2C8, 2C9, 2C19, 2D6, 2E1, and 3A4; the results of the study indicated IC_50_ values for chlorpyrifos, fenitrothion, and profennofos (4 μm), CYP2B6 (IC_50_ values of chlorpyrifos, fenitrothion, 2.5 μm), CYP2CB (fenitrothion, 4.3 μm), CYP2C9 (fenitrothion and malathion, 4.8 and 2.5 μm, respectively), and CYP2D6 (chlopyfifos and phenthoate-3 μm). Lo et al. [109] tested endocrine disrupters with antiandrogenic effects in vivo for their influence on 5a-reductase activity in two different test systems; the results indicated that the effect of the organotin compounds DBT (DIBUTYLTIN), TBT (tributyltin), and triphenyltin (TPT) on enzyme acitivity were almost the same in the two systems, with IC_50_ values ranging between 2.7 and 11.2 μm. Das et al. [77] determined the IC_50_ concentrations of the pesticides monocrotophos, chlorpyrifos, profenofos, and acephate as inhibitors of acetylcholinesterase (AChE); the results of the study indicated that the IC_50_ values for RBC-AChE were 0.12, 0.25, 0.35, and 4.0 μm for chlorpyrifos, monocrotophos, profenofos, and acephate, respectively. Zhang et al. [81] studied the relationship between liver conditions and neurodegenerative diseases and Butyrylcholinesterase (BChE); the results indicated accurate IC_50_ values for tacrine with respect to BchE (8.6 nm). Abdel-Halim et al. [8] discussed the ability to induce in vitro cytotoxic and oxidative stress in normal human cells with an MTT test; the results showed that the level of inhibition concentration (IC50) values was 0.023 and 0.025 mm for imidacloprid and glyphosate, respectively. Forsythe et al. [10] used bioengineered 3D human liver and cardiac organoids to screen a panel of thallium and glyphosate and discussed the response of the organoids to these compounds; the results indicated that the IC_50_ of glyphosate was 13.5 μm.

### 4.2. Chronic Disease

The likelihood of chronic health issues related to pesticide exposure is supported by a large number of data collected from laboratory animals [114,115,116]. However, epidemiological data are not available for all health issues. It has been documented that various chronic diseases and disorders occur after people have been exposed to pesticides [117,118,119], including cancers, adverse reproductive outcomes [120], male sterility [121], peripheral neuropathies [122], neurobehavioral disorders [123], impaired immune function (Nankongnab et al., 2020) [124], and allergic sensitization reactions, particularly of the skin [125,126,127]. Moreover, most of the pesticides examined affect male reproductive systems [124,125,126,127,128], causing sperm damage [118], DNA damage [129], and abnormal sperm morphology [13]. As an example, cumulative inhalation of cholinesterase activity as a result of long-term, low-dose exposure to organophosphorus compounds leads to chronic diseases [125]. Antonine et al. [121] tested the effects of low concentrations of Glyphosate; the decrease observed in levels of Clusterin mRNAs suggested that glyphosate targets the integrity of Sertoli cells. The decrease in the numbers of germ cells from day 14 onward highlighted the chronic effect of glyphosate at 50 nm, 500 nm, or 5 μm. Meltzer et al. [115] aimed to evaluate self-reported exposure to the Ringwood Mines/Landfill Superfund Site in relation to chronic health outcomes among members of the Ramapough Lunaape Turtle Clan nation and other residents of Ringwood, New Jersey. The results of the study indicated significant associations among Ringwood residents of Native American ethnicity between health issues and self-declared opportunities for Superfund site exposure. The results also showed a strong association between self-reported Superfund site exposure and the prevalence of bronchitis and asthma. Vanlaeys et al. [128] tested glyphostate alone, glyphosate-based herbside formulations, and POEA on an immature mouse Sertoli cell line (TM4) at concentrations ranging from environmental to agricultural-use levels; the results indicated that formulations of glyphosate-based herbicides induce TM4 mitochondrial dysfunction, disruption of cell detoxification systems, lipid droplet accumulation, and mortality at sub-agricultural doses. The results also showed that formulants, especially those present in Glyphogan, are more deleterious than glyphosate. Séralini et al. [78] discussed a 90-day feeding study which was conducted by Monsanto in order to achieve commercial release of this GMO, employing the sane rat strain and analyzing biochemical parameters in the animals. The results showed that in the treated males liver congestion and necrosis were 2.5 to 5.5 times higher than in the control groups. Marked and severe nephropathies were generally 1.3 to 2.3 times greater. In females, all treatment groups showed a two- to three-fold increase in mortality, and deaths occurred earlier. Males had more than four times the frequency of mammary tumors of the controls.

Research related to the impacts of the various routes of exposure on chronic disease are summarized below. Fang et al. [60] collected 300 samples from 8 main growing regions in China and detected pesticide residues. Both the chronic and acute intake risks of pesticides were assessed. Furthermore, intake risk for each detected pesticide was ranked according to a predefined ranking matrix. The results indicated that out of these 300 samples, 175 contained one or more pesticide residues. Twenty-five pesticides were identified in total, among which carbofuran was found to exceed the maximum residue limit. Chronic and acute intake risks were evaluated and were found to lie between 0 and 1.80 and between 0.05 and 28.0 for these 25 pesticides, respectively. The intake risks for individual pesticides were ranked. Five pesticides, including Avermectin, Triazophos, chlorpyrifos, dimethoate oxygen, and carbofuran, posed the highest risks. Tudi. et al. [12] discussed the potential chronic health risk of pymetrozine in soil and water in typical rice-growing areas of China; the results showed that the potential lifetime non-cancer risks associated with soil exposure for adults in both areas were higher than the potential non-cancer lifetime risks associated with dermal contact with paddy water. The potential non-cancer lifetime risks associated with soil exposure for adults in both areas were lower than the potential non-cancer lifetime risks associated with ingestion exposure to soil in both areas. Pan et al. [18] discussed the organophosphorus pesticides (OPPs) used on agricultural soils in the Yangtze River Delta of China; Dimethoate was found to be the primary compound, followed by methyl parathion and parathion. Soil ingestion was the primary exposure pathway of OPP exposure and contributed to 70–80% of the total risks. The non-cancer risks to children were relatively higher than the risks to adults. Yadav et al. [74] discussed the occurrence, distributions, and profile of selected OCP chemicals in surface soil samples from four major cities in Nepal; the results showed that soil ingestion was the main exposure route for the OPPs used in soil in Nepal in relation to cancer risk.

Three-million cases of severe acute poisoning may be matched by a greater number of unreported but mild cases of intoxication and acute conditions. Still, the numbers of cases of chronic effects are smaller than the number of acute effects [4]. The high levels of acute and chronic morbidity urgently require medical and rehabilitation services. An unknown number of less serious health effects would add to the overall disease burden, while precise estimation of these effects requires future epidemiological studies. Children, pregnant women, aging populations, and workers directly exposed to pesticides are at higher risk of being affected by pesticides and their related diseases [14,130,131].

## 5. The Impact of Pesticide Application Methods on Exposure and Health Risks Associated with Pesticide Use

Selecting the right equipment for appropriate pesticide application is important for application efficiency and minimization of the loss of spray solution [132], as well as the reduction of pesticide residuals in the environment and the exposure of and risks to residents and farmers [20,32,133]. Han et al. studied the effects of different spray equipment and formulations on the persistence of pyraclostrobin in *R. roxburghii*. The results indicated the following ordering: gaston gasoline piggyback agricultural sprayer (5.38 d) > manual agricultural backpack sprayer (3.37 d) > knapsack multi-function electric sprayer (2.91 d), suspension concentrate (SC) (6.78 d) > wettable powder (WP) (5.64 d) > water dispersible granule (WG) (4.69 d). Konthonbut et al. [27] discussed paraquat exposure among backpack sprayers in Thailand and analyzed the level of occupational exposure; the results of the study indicated that the use of battery-powered backpack sprayers and standing upwind effectively reduced inhalation exposure. Hunter et al. (2019 discussed the effect of different application speeds and nozzle types on the target area coverage and uniformity of UAV applications; the results of the study indicated that AIXR nozzles provided the best coverage among the nozzles tested and that they could reduce the risk of off-target movement. Li et al. [31] discussed the different operating parameters which could obtain a better reference for the determination of field operating parameters, and technical references for field pesticide application and the results of the study indicated that, compared to traditional spraying machinery, unmanned aerial sprayers had advantages of more uniform liquid deposition distribution and better penetration, providing a technical reference for field spray operations and the establishment of a uniform standard for pesticide application technology. Illyassou et al. [132] assessed the potential dermal exposure to pesticides associated with the use of hand-held and backpack sprayers; the results showed that exposure levels for operators using hand-held sprayers were higher than those for operators using backpack sprayers. Mahaboonpeeti et al. [53] assessed the potential exposure and risk levels associated with pesticides for farmers who used either a backpack sprayer with a two-stroke gasoline motor and fan or a battery-operated pump in Thailand; the results of the study showed that the estimated total body alachlor exposures of applicators using the two-stroke engine/fan backpack sprayer (219.48 μg/h) were significantly higher than those using the battery-operated pump backpack sprayer (15.50 μg/h). Lozier et al. [9] evaluated the occupational inhalation exposure to atrazine during pesticide application in a developing country; the results of the study indicated that tractor/boom pesticide application decreased overall population occupational exposure. Monitoring nozzles on booms from a distance rather than on the back of a tractor or a boom may decrease or eliminate inhalation exposure. The use of flat spray nozzles for herbicide application among pump backpack sprayers may also reduce their inhalation exposure.

## 6. General Health Problems Associated with Pesticide Exposure in Developing Countries

Even though the WHO and some developed nations have taken certain measures to reduce the negative impacts of pesticides, the serious problem of pesticide contamination in the environment and direct and indirect human exposure to pesticides is still a significant issue worldwide [14,44,45].

Although no segment of the general population is completely protected against exposure to pesticides and their potentially serious health effects, a disproportionate burden is shouldered by people in developing countries, as well as by high-risk groups in other countries [134,135,136]. For example, pesticide use causes three million poisonings, 220,000 deaths, and about 750,000 chronic illnesses every year worldwide, most of them occurring in developing countries [33]. Another example is the cost of pesticide-related diseases and harm caused in sub-Saharan Africa in 2005, which was found to be about USD 4.4 billion and was expected to increase to about USD 90 billion by 2020 [137]. A WHO (1990) report indicated that approximately 87 million people lived in Sichuan Province in China, which has an agricultural area of 6.5 million hectares. A total of 4 kg of pesticides was used per hectare, and 10 million people were exposed to these pesticides, with about 12% of the people poisoned. Moreover, the situation in developing countries has been changing rapidly, and, owing to climate change and the exponentially growing population, some new types of crops and other products require greater amounts of pesticides [33]. Thus, the percentage of people who are exposed to pesticides has been increasing.

Many reports about health problems are related to pesticide exposure in developing countries [138,139,140,141,142,143,144]. Regardless of the facts and the evidence of harm, most farmers in developing countries still use pesticides incorrect ways and at increasing rates, causing serious health effects [134,145,146]. The serious health problems related to pesticide exposure in developing countries occur because of insufficiently qualified institutions governing and evaluating their production and sales [14,147,148]. Moreover, developing countries lack strict laws and regulations that properly regulate pesticide exports and imports [125,149]. As an example, Atreya et al. [150] showed that it is crucial and necessary to set strict regulations to control farming practices in Nepal because farmers continue to buy highly toxic, obsolete pesticides. Furthermore, some highly toxic pesticides banned in developed countries are still being used there [127,134].

Intense usage by farmers, unsafe practices, and insufficient education about the use of pesticides are the main reasons for the general health problems in developing countries [151,152]. In addition, owing to large populations, farmers in developing countries are under increasing pressure to use pesticides to maintain their subsistence livelihoods [4,148]. Furthermore, farmers in developing countries do not have enough opportunities for education and training, hence they do not fully understand the chemical toxicity and the methods for the safe application of chemicals used in agricultural and household activities to control pests and diseases [153]. They also do not have enough power to control external forces, including market and trade liberalization and internal policies [127].

Another important reason for the general health problems in developing countries is that the public health systems of these nations do not have the capacity to adequately tackle pesticide-related health problems. This situation is made worse by many different types of pesticides in use that require different case management protocols [154]. According to previous studies, the use of pesticides will double in the next ten years in developing countries [14]. Agricultural practices continue to develop, so the number of cases of intentional and unintentional acute poisoning will likely increase. Organochlorine pesticides will be used less, but the use of insecticidal organophosphorus compounds and insecticidal carbamates is increasing [155]. The risks of acute intoxication will increase unless the use of most toxic pesticides is reduced [156]. Owing to the increase in cash-crops and plantation-style farming in developing countries, the number of individuals in high-risk occupations may also increase over the next decade [33].

In summary, when pesticides are applied to target plants, applicators and communities are directly exposed when they spray pesticides using various application methods and are indirectly exposed to pesticides through environmental contamination via oral, ingestion, and inhalation exposure routes, leading to acute toxic effects and chronic diseases. Therefore, it is necessary to assess exposure to and the human health risks associated with pesticides.

## 7. Methods of Health Risk Assessment Regarding Pesticide Application

### 7.1. Deterministic Risk Assessment

The deterministic risk assessment method is widely used in environmental and public health contexts [68,157,158,159,160,161,162,163,164]. It uses a single point to evaluate risk [68,165]. There are both advantages and disadvantages of deterministic risk assessment [166]. The advantage of the method is that it is easy to understand and conduct exposure and health risk evaluations of [158,167]. However, deterministic risk assessment only calculates average exposure and health risk. This kind of method does not consider the uncertainty of exposure and risk [165]. For example, the deterministic approach only shows the risk if the average value of exposure and the risk index is above or below a specified dose or standard of risk level. This is the case when the average exposure and health risk for one kind of chemical falls below the reference dose and standard of risk level; however, 20% of the population may exceed this dose. Based on a deterministic risk assessment, researchers may identify that there is no risk and thus no risk-management actions may be considered [168].

Previous studies that have assessed the exposure and health risk levels of pesticides by deterministic risk assessment are summarized in this paragraph. Lei et al. [59] assessed health risks due to exposure to organochlorine pesticides, polychlorinated biphenyls, polybrominated diphenyl ethers, polycyclic aromatic hydrocarbons, and toxic trace elements (mercury, chromium, cadmium, lead, and arsenic) based on animal-based foods collected from markets in Shanghai, China. The results showed that the combined hazard quotient values for multiple contaminants via single or multiple food consumption were below 1, suggesting that the residents in Shanghai would not experience a significant non-cancer health risk. Among the contaminants investigated, the highest potential non-cancer risk was associated with methylmercury. However, the combined cancer risk posed by multiple contaminants in most foods exceeded the accepted risk level of 10^−6^, and inorganic arsenic was the main contributor. Shi et al. [63] detected the pesticide level and evaluated its health risk level in marine edible fish samples which were collected from two important nearshore fishing sites in Shantou Harbor and Haimen Bay in eastern Guangdong Province, China. The results of the study showed that daily fish consumption in this region can be of serious concern, and lifetime cancer risk remains a possibility in the studied area. Siriwat et al. [57] assessed the health risks associated with skin exposure to chlorpyrifos and investigated the factors associated with chlorpyrifos exposure among children in agricultural areas of Sakon Nakhon Province, Northeastern Thailand. The results of the study showed that Chlorpyrifos residues were detected on the skin of 73.1% of the children tested. The health risk assessment of non-cancerous effects showed that children had dermal chlorpyrifos exposure levels of 1.46–10.5 mg/kg/day, and the HQ of child dermal chlorpyrifos exposure was 0.03, which is an acceptable level. Chidya et al. [166] employed an integrated approach combining monitoring and risk assessment of pesticides in Kurose River and its catchment area in Japan; the results showed that cyanazine had the highest detection level (64%), followed by simetryn (58%) and diazinon (57%), across all the sample sites (*n* = 12). Based on the HQ estimates, all the pesticides were below the threshold value of 1 and hence posed no significant health risks to humans. Wu et al. [158] studied the pesticide residual in honeysuckle in China and evaluated the potential health risks for consumers using the HQ and HI (hazard index) methods; the results of the study showed that the acute hazard quotient (HQa) of carbofuran was 1.54 for fetuses, infants, and pregnant or nursing women, which indicated that it posed a potential acute health risk. In the cumulative risk assessment, the acute hazard index (HIa) of insecticides in honeysuckle for children and the specific population were 1.34 and 3.36, respectively, suggesting that they posed potential acute cumulative health risks. Wang et al. [159] conducted seasonal and regional distribution assessments of 17 polycyclic aromatic hydrocarbons (PAHs) in surface waters from four different main water functional regions of Baiyangdian Lake; the results showed that the human health risk posed by PAHs in the surface water of Baiyangdian Lake did not constitute a potential non-carcinogenic risk to local residents and the carcinogenic risk was acceptable on the whole, though the potential lifetime carcinogenic risks for infants in rural residential regions should be a cause for concern. Urban residential regions and rural residential regions were subject to higher cumulative non-carcinogenic and carcinogenic risks when compared with other functional regions. Odewale et al. [68] detected residues of dichlorodiphenyltrichloroethanes (DDTs) and hexachlorocyclohexanes (HCHs) in forty-eight (48) composite fruit and vegetable samples (carrot, cucumber, tomato, and watermelon) and estimated average daily intake (EADI), cancer benchmark concentration (CBC), hazard quotient (HQ) and index (HI), and hazard ratio (HR). The results showed that the non-carcinogenic health risk of α-HCH in tomatoes and watermelon had a HQ > 1, which indicated the possibility of a systemic health risk for child consumers. The carcinogenic health risk showed that only α-HCH and γ-HCH in children and α-HCH in adults had a HR > 1 for tomato and watermelon, which implied the possibility of carcinogenic health risk from its consumption. Mercadante et al. [165] detected the concentration levels of pesticides in groundwater and estimated the health risk associated with drinking water for the adult population in Lombardy. The results indicated that about 1.5% determinations exceeded the environmental quality standard, but there was no potential health risk due to intake of contaminated groundwater detected for the general adult population. Simas et al. [38] investigated the prevalence of ventilatory dysfunction in workers and the characteristics of work in banana production in a region of the Ribeira Valley, Brazil. The prevalences of moderate obstructive disorder (10.0%), mild obstructive disorder (13.3%), and mild mixed disorder (3.3%) were determined. It was concluded that one-third of the workers had some type of ventilatory dysfunction, and a relationship was confirmed between ventilatory dysfunction, and the work involved in banana farming.

### 7.2. Probabilistic Risk Assessment

The probabilistic risk assessment method estimates the distribution of exposure and risk for a range of populations from lowest to highest risk [169]. There are three main advantages of probabilistic risk assessment. Firstly, probabilistic risk assessment can determine the proportion of the population that exceeds the specified reference dose, including the tolerable daily intake or risk-specific dose levels [170,171]. For example, if 20% of the population is exposed to one kind of chemical, the probabilistic risk assessment can determine that 20% of the population is at risk (Duan et al., 2021) [172]. Secondly, the results of probabilistic risk assessments have potential application in cost–benefit analyses [173]. A probabilistic risk assessment model can be run repeatedly, thereby providing various risk-management scenarios, e.g., removing contamination and recalling contaminated foods [174,175,176]. Thus, the necessary cost actions can be implemented to reduce these risk scenarios. Thirdly, probabilistic risk assessment allows for the estimation of uncertainty and unreliability [177,178].

#### 7.2.1. Risk Characterization and Quantification Using the Probabilistic Approach

Risk characterization and quantification using the probabilistic approach can be achieved through comparisons of the cumulative probability curves for exposure and effects [179]. The exposure curve is on the left-hand side of the effect curve, and the closeness or overlap between the two curves provides a qualitative indication of the relative degree of health risk [180]. A closer overlap between the two curves indicates that there is a higher risk. However, if the curves are far apart from each other and do not overlap, this indicates that the risk is low and should be negligible.

To obtain a quantitative comparison, a hazard quotient (HQ) needs to be calculated. For example, HQ_95/5_ is defined as the ratio of the exposure dose (or concentration) at 95% cumulative probability on the exposure curve to that at 5% cumulative probability on the effect curve [181]. A reference point of HQ_95/5_ = 1 corresponds to a risk probability of 1/20, or 5%. Similarly, a reference HQ_50/50_ = 1 represents a risk probability of 50%. This situation would also indicate that the two cumulative probability curves largely overlap with each other over the same dose intervals. However, while the HQ method is a simple method for risk quantification and comparison, the statistical distributions of the cumulative probability curves are not properly considered. The HQ may not indicate the real risk characteristics, especially when the distributions of exposure and effect data do not follow a normal distribution or have different slopes [182]. For example, Albert et al. (2018) considered the absorbed dose levels and consequent health risk associated with dermal exposure to chlorpyrifos among applicators on rice farms in Ghana. The results indicated that the median exposed group were at high risk of acute adverse health effects because of chlorpyrifos exposure, with hazard quotient (HQ_50_) values ranging from 1.5 to 5. In addition, the HQ_95_ values of 2.7 × 10^−9^ for the 5% highly exposed group represented a higher risk of acute adverse health effects. The chronic exposure guideline values further suggested that the 5% highly exposed group could be adversely affected due to chlorpyrious exposure, with HQ_95_ values ranging from 1.2 to 2. Phung et al. [77] evaluated chlorpyrifos exposure to rice farmers in Vietnam using a probabilistic approach; the results of the study indicated that the baseline exposure level, which ranged from 0.03 to 1.98 mg/kg/day, was below the chronic guideline level set by international and national bodies. However, the post-application exposure level, which ranged from 0.35 to 94 mg/kg/day, exceeded most of the acute guidelines in the 95th percentile.

#### 7.2.2. Monte Carlo Health Risk Assessment

By using the Monte Carlo technique, all variables and parameters which are used in risk assessment are regarded as a probability distribution [42,183,184]. The level of cancer risk and the hazard quotient are calculated 10,000 times or more with randomly chosen values of variables and parameters covering their range of variability [170,185] and reproducing the assumed distribution density [186,187,188]. The results of a Monte Carlo health risk assessment are provided in the form of a probability distribution for a given risk [172,189]. In addition, the results of Monte Carlo health risk assessment are useful for understanding the uncertainties related to interpersonal variability and biological factors and the dynamic character of contaminated media [64,185]. However, Monte Carlo simulation requires two cumulative probability distributions, and this restricts the application of the method [177].

A number of Monte Carlo health risk assessments in relation to pesticide exposure and human health risk have been conducted. Faezeh et al. (2021) [189] detected concentrations of 18 organophosphorus, carbamate, pyrethroid, and nicotinoid pesticides in six walnut cultivars from five geographical regions in Iran. Human health risk assessments were evaluated using hazard indices (HIs) with Monte Carlo simulations. The 95th percentile of HIs for humans based on exposure via ingestion of walnuts was estimated to be 1.68, which represented a moderate concern for human consumers. The most influential parameters, determined by sensitivity analysis conducted during the MCS, were concentrated, and ranged from 0.71 to 0.97. The results showed that the walnuts were, in general, safe to eat. Korucu et al. [42] evaluated the environmental risks of p,p′-DDT and its metabolites (DDX) on human and terrestrial species through exposure to soil and agricultural products in agricultural areas in Turkey. Using the Monte Carlo simulation method, the intake values were calculated for five different exposure groups, four different bird species, and four different mammal species. The results showed that there was a high level of carcinogenic risk in humans. Furthermore, a significant risk of reproductive toxicity was determined for birds and mammals. The main source of the risk was exposure to DDX-contaminated soils and the consumption of plants grown on these soils. Eslami et al. [175] evaluated the hazard quotient and total hazard quotient (THQ) in a human health risk assessment for pesticides in fruit grown in Iran using the Monte Carlo approach and the results showed that THQ in adults based on consumption of fruits was estimated to be 7.8% and 36.7% for adults and children, respectively. Thus, there was no risk to human health for adults and children. Atabila et al. [170] assessed health risks associated with chlorpyrifos exposure among applicators on rice farms in Ghana by means of advanced probabilistic approaches; the results showed that the probabilities of adverse health effects due to chlorpyrifos occurring under the chronic exposure scenarios ranged from 1 to 8%, while those for acute exposure scenarios ranged from 31 to 34%. Thus, there are both chronic and acute human health effects from chlorpyrifos exposure among applicators in Chana. Pirsaheb et al. [185] detected residues of pyrethroid and organophosphorus pesticide in flour and breads which were collected from local markets in Kermanshah Province, Iran, and assessed the health risk of these pesticides for adults and children using the Monte Carlo simulation method. The results of the study showed that 15% and 11.1% of total samples contained detectable levels of deltamethrin and malathion, respectively. About 85% of pesticide residue detections were observed in tropical and mild weather areas due to high consumption rates of insecticides. For both deltamethrin and malathion in adults and children, the 95th percentile of the THQ value was lower than 1 (i.e., an acceptable level); thus, there was no non-carcinogenic health risk due to deltamethrin and malathion detected for bread consumers in Kermanshah Province.

#### 7.2.3. Overall Risk Probability Health Risk Assessment

The overall risk probability (ORP) health risk assessment method provides a simple probability measure, which allows an easy comparison of the relative risks of individual compounds [170]. Furthermore, it is a multiple-point method, as the statistical distributions of both the exposure and effect curves are considered correctly [177]. Moreover, the ORP method can quantify the combined effects of either independent or interacting factors [181]. However, this method requires the construction and integration of an exposure exceedance curve to obtain the ORP and it may require more computational resources compared to single-point methods [190]. For example, Atabila et al. [170] assessed the exposure and health risk of chlorpyrifos in Ghana using the overall risk probability risk assessment method. The results indicated the probabilities of adverse effects among applicators due to chlorpyrifos from chronic background exposure (3%), chronic exposure from occupational application (4%), and acute exposure from occupational application (31%). Phung et al. [177] assessed the exposure and health risk posed by chlorpyrifos for Vietnamese rice farmers in an overall risk assessment and the results showed the probabilities of adverse effects among applicators due to chlorpyrifos from chronic background exposure (1%), chronic exposure from occupational application (2%), and acute exposure from occupational application (29%).

## 8. Conclusions and New Directions

### 8.1. Conclusions

There are four main pesticide application methods that have been evaluated in this study, including hydraulic spraying, backpack spraying, basal trunk spraying, and aerial spraying. Equipment for appropriate pesticide application is important for application efficiency to minimize the loss of spray solution and reduce pesticide residuals in the environment and adverse human health effects due to over-spraying and residues. When pesticides are applied to target plants, applicators and communities are directly and indirectly exposed to pesticides through oral, ingestion, and inhalation routes, resulting in acute effects and chronic diseases. Therefore, it is necessary to conduct exposure assessments and human health risk evaluations in relation to pesticide applications.

The human health risk assessment framework includes hazard identification, exposure assessment, and dose–response assessment and risk characterization. There are several advantages to using probabilistic risk assessment methods. (1) Probabilistic risk assessment can determine the proportion of a population that exceeds the specified reference dose, including tolerable daily intake and risk-specific dose levels. (2) Probabilistic risk assessment models can be run repeatedly, thus providing various risk-management scenarios. (3) Probabilistic risk assessment provides the opportunity to estimate uncertainty and unreliability. However, the probabilistic risk assessment process is more complicated and needs large sets of data, and it is not easy to estimate risks for new chemicals.

### 8.2. New Directions

Both applicators and residents may easily ignore long-term pesticide exposure through multiple exposure routes, and this can result in serious health problems. Thus, further studies should focus on both occupational and environmental exposure and related health risk assessments for pesticides considering multiple exposure routes to ensure better pesticide use and management in the future. In addition, it is crucial to convey the scientific outcomes of exposure and occupational and environmental health risk assessments and to provide scientific training for the application of pesticides, the prevention of adverse health effects due to pesticide usage, and the promotion of the health of applicators and communities in order to support sustainable development. As members of communities who are directly exposed to pesticides, farmers should possess sufficient knowledge, adopt proper attitudes, and correct perceptions concerning the use of pesticides so as to reduce human health risks. Policymakers should implement several useful measures, such as integrated pest management (IPM), laws that prohibit the use of pesticides with high health risks, and the development of a national implementation plan (NIP) to reduce the adverse effects of pesticides on the environment and human health. These approaches are more eco-friendly and aim at controlling the negative influence of pesticides on the environment and human health. Policymakers should also pass laws to ban or restrict the use of pesticides that are highly toxic or hazardous to the environment and human health. Furthermore, policymakers should set strict regulations for farmers regarding the use pesticides at the recommended doses.

## Data Availability

Not applicable.
